# Molecular Evolution and Stress and Phytohormone Responsiveness of *SUT* Genes in *Gossypium hirsutum*

**DOI:** 10.3389/fgene.2018.00494

**Published:** 2018-10-23

**Authors:** Wei Li, Kuan Sun, Zhongying Ren, Chengxiang Song, Xiaoyu Pei, Yangai Liu, Zhenyu Wang, Kunlun He, Fei Zhang, Xiaojian Zhou, Xiongfeng Ma, Daigang Yang

**Affiliations:** ^1^State Key Laboratory of Cotton Biology, Institute of Cotton Research of Chinese Academy of Agricultural Sciences, Anyang, China; ^2^College of Agriculture, Yangtze University, Jingzhou, China

**Keywords:** cotton, sucrose transporter, phylogenetic relationship, expression profile, abiotic stress, phytohormone

## Abstract

Sucrose transporters (SUTs) play key roles in allocating the translocation of assimilates from source to sink tissues. Although the characteristics and biological roles of *SUTs* have been intensively investigated in higher plants, this gene family has not been functionally characterized in cotton. In this study, we performed a comprehensive analysis of *SUT* genes in the tetraploid cotton *Gossypium hirsutum*. A total of 18 *G. hirsutum SUT* genes were identified and classified into three groups based on their evolutionary relationships. Up to eight *SUT* genes in *G. hirsutum* were placed in the dicot-specific SUT1 group, while four and six *SUT* genes were, respectively, clustered into SUT4 and SUT2 groups together with members from both dicot and monocot species. The *G. hirsutum SUT* genes within the same group displayed similar exon/intron characteristics, and homologous genes in *G. hirsutum* At and Dt subgenomes, *G. arboreum*, and *G. raimondii* exhibited one-to-one relationships. Additionally, the duplicated genes in the diploid and polyploid cotton species have evolved through purifying selection, suggesting the strong conservation of *SUT* loci in these species. Expression analysis in different tissues indicated that *SUT* genes might play significant roles in cotton fiber elongation. Moreover, analyses of *cis*-acting regulatory elements in promoter regions and expression profiling under different abiotic stress and exogenous phytohormone treatments implied that *SUT* genes, especially *GhSUT6A/D*, might participate in plant responses to diverse abiotic stresses and phytohormones. Our findings provide valuable information for future studies on the evolution and function of *SUT* genes in cotton.

## Introduction

Sucrose, the major form of sugar transported in plants, is allocated to various sink tissues to support growth and storage. Sucrose transporters (SUTs also called SUCs) play important mediating roles in the active transport of sucrose across the plasma membrane from source tissue to some sink cells Thomasl and Davidm (2010). Plant SUTs belong to the major facilitator superfamily (MFS), which is characterized by the presence of 12 predicted transmembrane-spanning domains with N- and C- termini in the cytoplasm (Lalonde et al., 2004; Sun et al., 2010). The first identified plant *SUT* gene, *SoSUT1*, was isolated from spinach by yeast functional complementation (Riesmeier et al., 1992). A large number of SUT proteins have subsequently been identified in many plant species, including monocots such as rice (Aoki et al., 2003), maize (Usha, 2015), sorghum (Milne et al., 2013), and hexaploid wheat (Deol et al., 2013), and dicots such as Arabidopsis (Weise et al., 2000), *Populus* (Hackel et al., 2006; Payyavula et al., 2011), cacao (Li et al., 2014b), and pear (Zhang et al., 2013). These findings have revealed that *SUT* is a small gene family usually comprising three to nine members per species.

On the basis of phylogenetic and structural analyses, plant SUTs have been divided into five subgroups: SUT1 (dicot specific), SUT3 and SUT5 (monocot specific), and SUT2 and SUT4 (both monocots and dicots). *SUT* family genes are not only involved in sucrose transport, but also play essential roles in pollen germination, fruit ripening, and ethylene biosynthesis in many species (Sivitz et al., 2008; Srivastava et al., 2009; Payyavula et al., 2011; Chincinska et al., 2013). For example, the *AtSUC1* gene in *Arabidopsis thaliana* is predominantly expressed in pollen and facilitates anthocyanin accumulation (Sivitz et al., 2008). Another Arabidopsis gene, *AtSUC5*, has specific expression in seeds (Baud et al., 2005), and the mutant *atsuc9* functions to promote the floral transition by regulating the uptake of sucrose (Sivitz et al., 2007). In rice, disruption of the expression of the *OsSUT1* gene impairs pollen function (Hirose et al., 2010). The *OsSUT2* gene is significantly expressed in germinating embryos of rice seeds (Siao et al., 2011; Eom et al., 2016). In maize, a *SUT1* mutant displays growth retardation and hindered tassel development (Usha, 2015). In *Populus*, *PtaSUT4*-RNA interference (RNAi) plants have higher shoot water contents and delayed wilting relative to wild-type plants (Frost et al., 2012). In hexaploid wheat, the *TaSUT2* gene is expressed in the veins of developing seeds and the subepidermal mesophyll cells of leaf blades (Deol et al., 2013). In transgenic tomato, independent inhibition of the expressions of *LeSUT1* and *LeSUT2* affects fruit and seed development (Hackel et al., 2006).

The genus *Gossypium* (cotton) consists of 50 species, of which 45 are diploid (2*n* = 2*x* = 26) and five are tetraploid (2*n* = 4*x* = 52) species. The genomes of diploid cotton species are labeled as followes: A, B, C, D, E, F, G, and K (Wendel and Cronn, 2002). The tetraploid cotton *Gossypium hirsutum* (upland cotton), the most extensively cultivated cotton species, is one of the most economically important crops in the world and a naturally renewable fiber source for the textile industry (Ulloa et al., 2005; Pang et al., 2010). Thus far, however, the functions of *SUT* genes in cotton are largely unknown, especially those in response to abiotic and biotic stresses. The recent completion of genome sequencing of *G. hirsutum* provides an opportunity to systematically analyze the *SUT* gene family in cotton.

In this study, we carried out genome-wide identification of the *SUT* gene family in *G. hirsutum* and analyzed phylogenetic relationships, gene structures, chromosomal locations, and collinearity. We then investigated putative *cis*-elements related to stress responses in the promoters of *G. hirsutum SUT* genes. Finally, we used real-time quantitative PCR (qRT-PCR) to profile *SUT* gene expressions in different tissues and under various abiotic stress conditions, including cold, heat, drought, and salt, and under hormonal stresses, including auxin (IAA), gibberellin (GA), and salicylic acid (SA). Our findings provide valuable insights into the evolution, expansion, tissues-specific expression, and stress responses of *SUT* genes in cotton.

## Materials and Methods

### Identification and Sequence Analysis of *SUT* Genes

Genomic and protein sequences of *G. arboreum*, *G. raimondii*, and *G. hirsutum* were downloaded from https://www.cottongen.org. We also downloaded protein sequences for the following species: rice (v7.0^1^), sorghum (v3.1.1^2^), *Brachypodium distachyon* (v3.1^3^), cacao (v2.0^4^), grape (12X^5^), and tomato (v3.2^6^). Amino acid sequences of *A. thaliana* SUTs were acquired from the TAIR 10 database^7^ and used as queries to search the *G. hirsutum* genome database with the BlastP program. All candidate *G. hirsutum SUT* sequences were then filtered to confirm the presence of the MFS domain using Pfam database analyses (*E*-value cut-off of 1.0^8^). To verify sequence accuracy, the putative *SUT* genes were subsequently aligned, and those with inconsistent alignments were cloned to determine the complete sequences. *SUT* genes in rice, maize, sorghum, *B. distachyon*, cacao, grape, tomato, *G. arboreum*, and *G. raimondii* were analyzed as described above for *G. hirsutum*. Finally, the molecular weight (MW) and isoelectric point (pI) of each deduced *G. hirsutum* SUT proteins was predicted using DNAMAN v9.0 software.

### Multiple Sequence Alignments and Phylogenetic Analysis

The complete protein sequences of SUTs were comprehensively aligned using MegAlign 7.1.0 with default settings (Clewley and Arnold, 1997). Phylogenetic analysis of the full-length SUT protein sequences of *A. thaliana*, rice, sorghum, *B. distachyon*, cacao, grape, tomato, *G. arboreum*, *G. raimondii*, and *G. hirsutum* was carried out using neighbor joining as implemented in MEGA (v7.0) (Tokyo Metropolitan University, Tokyo, Japan). A bootstrap analysis was performed with 1,000 iterations.

### Gene Structure, Chromosomal Localization, and Gene Duplication Analysis

The exon/intron organizations of *G. hirsutum SUT* genes were obtained by comparing their CDS sequences and corresponding genomic sequences using the GSDS server (Guo et al., 2007).

*SUT* gene loci were extracted from the genome annotation gff3 file. Orthologous and paralogous groups of the *SUT* gene family among the three *Gossypium* species were identified on the basis of phylogenetic trees and sequence alignments (Wang et al., 2014; Cui et al., 2017). To identify segmental duplications in diploid and polyploid cotton species, all SUT protein sequences were queried against a local database using the BlastP program and analyzed with MCScanX^9^ (Wang et al., 2013). Finally, the resulting collinearity map was plotted using Circos (Krzywinski et al., 2009).

### Calculation of Ka/Ks Values of Duplicated Genes

The gene coding sequences from segmentally duplicated pairs and orthologous pairs were primarily aligned using paraAT2.0 (Zhang et al., 2012); thereafter, the aligned sequences were employed to estimate non-synonymous (Ka) and synonymous (Ks) substitution rates with KaKs_Calculator2.0 (Zhang et al., 2006). The calculated Ka/Ks ratios were then analyzed to explore the selection pressure on each duplicated gene pair. Generally, a Ka/Ks ratio greater than, equal to, or less than 1 indicates positive (diversifying) selection, neutral evolution, or purifying (negative) selection, respectively. The divergence time *t* of each gene pair was subsequently estimated using the formula *t* = Ks/2λ, with λ, the neutral substitution rate, set to 2.6 × 10^-9^ (Senchina et al., 2003; Zhang et al., 2015).

### Plant Materials and Stress and Hormone Treatments

*Gossypium hirsutum* cultivar TM-1 was used in this study. After germination on wet filter paper for 3 days at 28°C, cotton seedlings were transferred to liquid medium containing nutrients and grown in a plant growth chamber under a 16-h light/8-h dark cycle for 2 weeks. Three-leaf-stage seedlings were subjected to one of several different treatments. To explore response to temperature stress, seedlings were incubated at 4 and 38°C in a lighted growth chamber, respectively. For drought and salt treatments, the roots of cotton seedlings were submerged in a solution containing 20% PEG 6000 and 150 mM NaCl. The leaves of treated plants were harvested at 0, 1, 3, 6, and 12 h. For GA, SA, and IAA treatments, seedlings were irrigated with 100 μM GA, SA, or IAA, respectively, and their roots were sampled at 0, 0.5, 1, 3, and 5 h. All harvested samples were immediately frozen in liquid nitrogen and kept at -80°C for total RNA isolation.

### RNA Extraction and qRT-PCR Analysis

A plant RNA purification kit (Tiangen, Beijing, China) was used to extract RNA from different organs and stress-treated samples. DNase I was then used to remove any genomic DNA contamination from the extracted RNA. The RNA concentration in each sample was measured by 1.5% gel electrophoresis and on a Nanodrop2000 nucleic acid analyzer. For each sample, the first cDNA strand was synthesized from 1 μg total RNA using a PrimeScript RT reagent kit (Takara, Dalian, China). The cDNA was diluted fivefold for subsequent experiments. Homologous gene-specific primer pairs for real-time PCR were designed using Primer-BLAST^10^ and are listed in Supplementary Table S1. The *G. hirsutum Histone3* gene, *GhHis3*, was used as an internal control. Transcript levels were determined by qRT-PCR using a LightCycler480 96 system (Roche, Mannheim, Germany) and SYBR Premix Ex *Taq* (2 × ) (Takara). PCR amplification parameters were as follows: 95°C for 30 s, followed by 40 cycles of 95°C for 5 s, 60°C for 1 min, and 72°C for 10 s, with a final step of 50°C for 30 s. The gene expression data were calculated using the 2^-ΔΔCt^ method (Bustin and Mueller, 2005). Finally, the data were plotted with Origin 9 software.

## Results

### Identification of *SUT* Genes in *G. hirsutum*

To globally identify members of the *SUT* gene family in cotton, we performed BlastP searches of nine Arabidopsis SUT proteins against the *G. hirsutum* genome database. As a result, 18 deduced *SUT* genes were identified in upland cotton. Following multiple sequence alignment, the sequences of two candidate genes were cloned and then manually edited. All putative SUTs were further verified using InterProScan to confirm the existence of the highly conserved MFS domain, thereby confirming the presence of 18 typical *SUT* genes in the entire genome of *G. hirsutum*. For comparative purposes, nine *SUT* genes each were also identified in the genomes of two diploid cotton species, *G. raimondii* and *G. arboreum* using the same methods. *SUTs* in *G. raimondii* were named *GrSUT1–GrSUT9* according to their genomic locations and *SUT* genes in *G*. *arboreum* were then assigned names based on their homologs in *G. raimondii* (Supplementary Table S2). Finally, *SUT* genes in *G. hirsutum* were given names corresponding to their orthologs in *G. raimondii* and *G. arboreum*, with suffixes D and A appended after each gene names to, respectively, indicate the Dt and At subgenomes. Characteristics of the identified *G. hirsutum SUT* genes, including gene name, ID, protein length, MW, pI, and chromosomal location, are detailed in Table 1.

**Table 1:** Characteristics of *G. hirsutum SUT* genes.

Gene name	Locus ID	Protein length (aa)	MW (kDa)	pI	Strand	Chromosome	Location
*GhSUT1A*	Gh_A02G0530	498	52.74	8.62	Minus	A02	7,947,539–7,952,892
*GhSUT1D*	Gh_D02G0595	498	52.57	7.94	Minus	D02	8,087,060–8,089,217
*GhSUT2A*	Gh_A02G0626	494	52.60	9.08	Minus	A02	9,893,346–9,895,488
*GhSUT2D*	Gh_D02G2392	494	52.61	8.80	Plus	scaffold3787_D02	4,634–6,739
*GhSUT3A*	Gh_A03G0862	616	65.61	6.50	Minus	A03	50,073,755–50,079,126
*GhSUT3D*	Gh_D02G1242	616	65.73	6.27	Minus	D02	39,707,577–39,712,897
*GhSUT4A*	Gh_A12G1524^a^	626	67.60	8.00	Plus	A12	74,966,271 –74,971,593
*GhSUT4D*	Gh_D12G1646	615	66.02	7.49	Plus	D12	48,002,866 –48,008,172
*GhSUT5A*	Gh_A05G3779	506	54.64	8.38	Minus	scaffold1220_A05	2,662–11,096
*GhSUT5D*	Gh_D05G2074	506	54.71	8.38	Plus	D05	19,223,532–19,231,507
*GhSUT6A*	Gh_A05G2131	558	59.10	8.34	Minus	A05	24,249,084–24,252,012
*GhSUT6D*	Gh_D05G2381	549	58.14	8.27	Plus	D05	23,730,693–23,733,726
*GhSUT7A*	Gh_A06G0230	507	54.62	8.55	Plus	A06	2,713,050–2,717,707
*GhSUT7D*	Gh_D06G2313	507	54.63	8.55	Plus	scaffold4091_D06	152,064–156,753
*GhSUT8A*	Gh_A13G0640	617	66.02	7.48	Minus	A13	17,357,241–17,361,898
*GhSUT8D*	Gh_D13G0757	617	65.83	6.86	Minus	D13	12,199,245–12,203,868
*GhSUT9A*	Gh_A13G1022	500	53.43	8.55	Minus	A13	57,060,564–57,063,716
*GhSUT9D*	Gh_D13G1273	500	53.34	8.41	Minus	D13	39,078,454–39,081,595


*^*a*^Gene coding sequences were manually re-annotated.*

### Sequence Alignment and Phylogenetic Analysis of *SUT* Genes in *G. hirsutum*

To explore the properties of SUT proteins, we aligned the amino acid sequences of the 18 *G. hirsutum* SUT proteins. As revealed by the multiple sequence alignment, all 18 contained 12 transmembrane helix domains. Moreover, the SUTs had consensus sequences that were highly conserved, including the histidine residue and other motifs in the extra cellular loop (Supplementary Figure S1). The conserved histidine residue is involved in sucrose binding during the transport process. A dileucine motif (LRQLX) was observed in the cytoplasmic N-terminus of proteins in the SUT4 group (GhSUT5A, GhSUT5D, GhSUT7A, and GhSUT7D).

**FIGURE 1:**
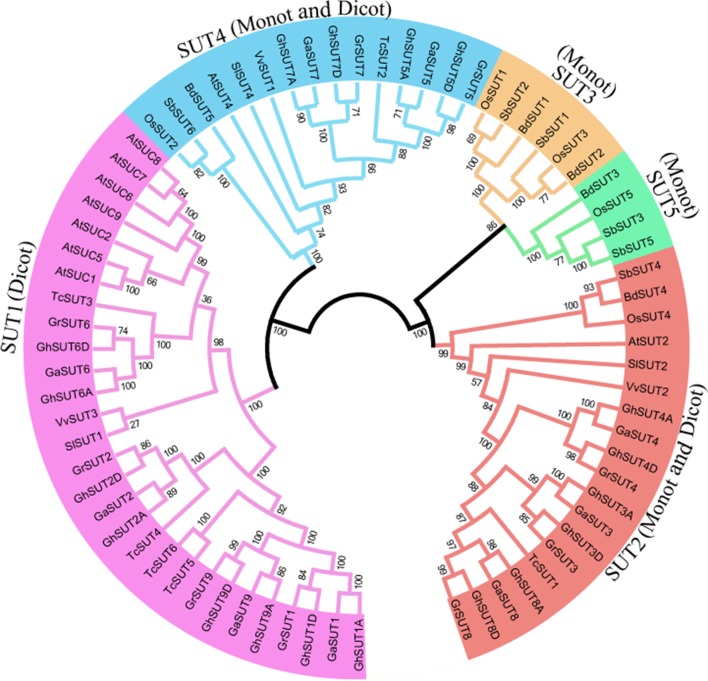
Neighbor-joining phylogenetic tree of SUT proteins from three *Gossypium* species and other plants SUT homologs. The SUT proteins were classified into five groups, which are shown in different colors. Loci and numbers of different plant SUT groups are listed in Supplementary Tables S3, S4, respectively.

To systematically infer evolutionary relationships of *SUT* genes between *G. hirsutum* and other plant species, the full-length sequences of *SUT* genes in three *Gossypium* species and other representative plants, including three monocot and four dicot species, were used to generate a phylogenetic tree. In the resulting tree (Figure 1), all 73 identified SUT proteins were divided into five classes (SUT1, SUT2, SUT3, SUT4, and SUT5). Among the five groups, SUT1 genes were specific to dicotyledonous species, while SUT3 and SUT5 genes were restricted to monocots. SUT2 and SUT4 included both dicots and monocots. Notably, the *SUT* gene family comprised two distinct clades in the phylogenetic tree, with SUT1 and SUT4 in one clade and the other three groups in another, thus indicating that two ancestral SUTs were present in both dicots and monocots.

In dicot-specific SUT1, closely related paralogs were present in each dicotyledonous species, which suggests that gene duplication events contributed to the amplification of the *SUT* gene family after the divergence of dicots from monocots. Interestingly, SUT2 and SUT4 had only one representative in each dicot and monocot species except for the three *Gossypium* species In contrast, three *G. raimondii*, three *G*. *arboreum*, and six *G*. *hirsutum* SUT2 genes were detected, with two *G. raimondii*, two *G*. *arboreum*, and four *G*. *hirsutum* genes belonging to SUT4. The presence of these multiple copies provides further evidence of the expansion of SUT2 and SUT4 gene groups in cotton. In monocot-specific SUT3 and SUT5, recent gene duplication events were inferred to have taken place in sorghum.

### Chromosomal Locations and Syntenic Analysis of *SUT* Genes in *G. hirsutum*

**FIGURE 2:**
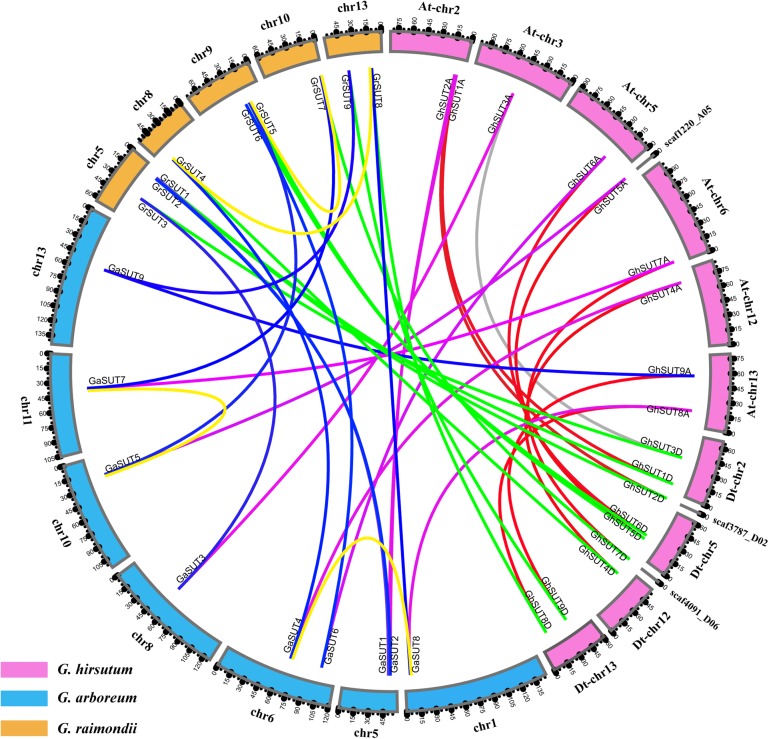
Locations and syntenic relationships of *SUT* genes from *G. hirsutum, G. arboreum* and *G. raimondii*. Chromosomes of *G. hirsutum, G. arboreum* and *G. raimondii* are indicated by pink, blue, and yellow, respectively. Putative homologous *SUT* genes between homoeologous chromosomes of *G. hirsutum* At and Dt subgenomes, the At subgenome and *G. arboreum*, the Dt subgenome and *G. raimondii*, and *G. arboreum* and *G. raimondii* are connected by red, purple, green and blue, respectively. Gray line link homologous genes located on non-homoeologous chromosomes in the At and Dt subgenomes. Duplicated gene pairs in *G. arboreum* and *G. raimondii* are connected by yellow lines.

*Gossypium hirsutum* (AD1), an allotetraploid species, was ultimately derived from the natural hybridization of two diploid species resembling *G. arboreum* (A2) and *G. raimondii* (D5) followed by chromosome doubling and natural and human selection. When referring to the genomic composition of this species, chromosome numbers 1–13 are reserved for the A subgenome (At), while chromosome numbers 14–26 are assigned to the D subgenome (Dt) (where ‘t’ indicates tetraploid) (Li et al., 2015). To better understand the physical distribution and collinear relationships of intraspecific and interspecific homologous genes among the three *Gossypium* species, we investigated *SUT* gene loci on cotton chromosomes and performed a synteny analysis (Figure 2 and Supplementary Figure S2). In *G. arboreum*, the nine *SUT* genes were distributed unevenly on seven chromosomes. Chromosomes 5 and 6 harbored two genes each. The other five genes were located on chromosomes 1, 8, 10, 11, and 13. In *G. raimondii*, the nine *SUTs* were mapped on five chromosomes, with chromosome 5 containing the largest number of genes. Two genes were present on chromosomes 9 and 13, while only one *SUT* gene each was located on chromosomes 8 and 10. In addition, 15 of the 18 *G. hirsutum SUT* genes were present on 8 of 13 At chromosomes and 4 of 13 Dt chromosomes. Three genes (*GhSUT2D*, *GhSUT5A*, and *GhSUT7D*) were distributed on scaffolds whose exact locations on chromosomes were not determined. Chromosomes At-chr2, At-chr13, Dt-chr2, Dt-chr5, and Dt-chr13 contained two genes each, while one gene each was present on chromosomes At-chr3, At-chr5, At-chr6, At-chr12, and Dt-chr12.

The collinearity analysis revealed one-to-one relationships between homologous genes of the two diploid cotton species and the two subgenomes of the tetraploid species. In addition, most *SUT* loci were significantly conserved. For example, nine *SUT* paralogous gene pairs were identified between the At and Dt subgenomes of *G. hirsutum*, eight of which were anchored across five homoeologous chromosomes (Supplementary Figure S2). Only the paralogous pair *GhSUT3A* and *GhSUT3D* was located on the non-homoeologous chromosomes, possibly the result of a chromosomal translocation. Consistently, all nine *G. arboreum SUTs* and *G. raimondii SUTs* had corresponding orthologous genes in the *G. hirsutum* genome. We also investigated gene duplication events in the three *Gossypium* species. Two segmental duplication genes pairs were identified in *G. arboreum* (*GaSUT4* and *GaSUT8*, *GaSUT5* and *GaSUT7*) and *G. raimondii* (*GrSUT4* and *GrSUT8*, *GrSUT5* and *GrSUT7*), respectively (Figure 2). Notably, they belonged to the same subfamilies (SUT2 and SUT4 groups) and were also orthologous genes between the two diploid cotton. This result indicates that segmental duplications in SUT2 and SUT4 groups may have contributed to the amplification of the *SUT* gene family in *G. arboreum* and *G. raimondii*. Natural polyploidization then doubled the number of *SUTs* during the subsequent evolution of *G. hirsutum*. Whereas, no tandem duplication event was observed.

To assess the molecular evolutionary history of *SUT* genes in cotton, we next calculated the Ka/Ks ratio of each duplicated gene pair between both diploid and polyploid cotton species (Table 2 and Supplementary Table S5). Almost all calculated Ka/Ks values (except for *GaSUT3/GhSUT3A*, and *GaSUT7*/*GhSUT7A*) in inter-genomic (At and Dt) and intra-genomic (A2 and At or D5 and Dt) comparisons were less than 1; this demonstrates that these genes have undergone strong purifying selection pressure after duplication, which could lead to limited functional divergence followed by segmental duplications and polyploidization. Only two gene pairs (*GaSUT3/GhSUT3A*, and *GaSUT7*/*GhSUT7A*) have Ka/Ks larger than 1, indicating that these genes might have experienced relatively rapid evolution following duplication. We also estimated divergence time of duplicated *SUT* gene pairs in the diploid cotton (Supplementary Table S5). In *G. arboreum* and *G. raimondii*, segmental duplications were estimated to have occurred between 59.519 and 74.981 million years ago (MYA). These observations provide insights into the evolutionary conservation of the *SUT* gene family between the *G. hirsutum* genome and the two diploid genomes.

**Table 2:** Ka and Ks values of duplicated *SUT* genes between both diploid and polyploid cotton species.

Homologous pairs	Identities (%)	Ka	Ks	Ka/Ks	Purifing selection
*GaSUT1/GhSUT1A*	98.94%	0.0045	0.0136	0.3292	Yes
*GaSUT2/GhSUT2A*	98.93%	0.0018	0.0166	0.108	Yes
*GaSUT3/GhSUT3A*	98.76%	0.0094	0.0089	1.0468	No
*GaSUT4/GhSUT4A*	95.85%	0.0542	0.0764	0.7099	Yes
*GaSUT5/GhSUT5A*	99.01%	0.0044	0.0181	0.2451	Yes
*GaSUT6/GhSUT6A*	99.52%	0.0008	0.0166	0.0484	Yes
*GaSUT7/GhSUT7A*	99.61%	0.0018	0.0000	99.057	No
*GaSUT8/GhSUT8A*	99.51%	0.0021	0.0044	0.4854	Yes
*GaSUT9/GhSUT9A*	99.87%	0.0009	0.0027	0.3275	Yes
*GrSUT1/GhSUT1D*	98.93%	0.0072	0.0217	0.3303	Yes
*GrSUT2/GhSUT2D*	99.46%	0.0018	0.0166	0.1082	Yes
*GrSUT3/GhSUT3D*	99.19%	0.0036	0.0089	0.4004	Yes
*GrSUT4/GhSUT4D*	99.19%	0.0036	0.009	0.3977	Yes
*GrSUT5/GhSUT5D*	99.60%	0.0018	0.0052	0.3438	Yes
*GrSUT6/GhSUT6D*	99.70%	0.0008	0.0096	0.0845	Yes
*GrSUT7/GhSUT7D*	99.61%	0.0018	0.0052	0.3424	Yes
*GrSUT8/GhSUT8D*	99.03%	0.005	0.0179	0.2789	Yes
*GrSUT9/GhSUT9D*	99.73%	0.0009	0.0081	0.109	Yes
*GhSUT1A/D*	96.59%	0.0153	0.0385	0.3979	Yes
*GhSUT2A/D*	98.79%	0.0054	0.0422	0.1276	Yes
*GhSUT3A/D*	96.27%	0.0199	0.0469	0.4245	Yes
*GhSUT4A/D*	92.82%	0.0245	0.0281	0.8738	Yes
*GhSUT5A/D*	98.22%	0.008	0.0396	0.2022	Yes
*GhSUT6A/D*	97.67%	0.0033	0.0343	0.0952	Yes
*GhSUT7A/D*	99.01%	0.0044	0.0315	0.1405	Yes
*GhSUT8A/D*	97.73%	0.1079	0.0457	0.2365	Yes
*GhSUT9A/D*	98.40%	0.0071	0.0191	0.3724	Yes


### Analysis of Exon/Intron Organization of *SUT* Genes in *G. hirsutum*

**FIGURE 3:**
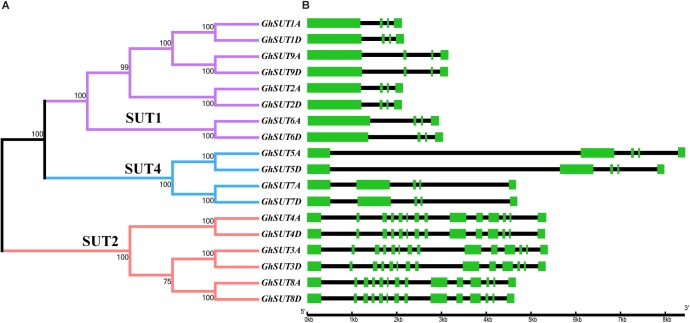
Phylogenetic relationships and structure of *G. hirsutum SUT* genes. **(A)** Neighbor-joining phylogenetic tree of *G. hirsutum* SUT proteins. The *SUT* genes in *G. hirsutum* were classified into three groups, SUT1, SUT4, and SUT2, which are represented by purple, blue, and orange, respectively. **(B)** Exon–intron structures of *SUT* genes. Green boxes and black lines represent exons and introns, respectively.

The arrangement of exons and introns can be used to analyze the evolution of gene families. To shed light on the structural evolution of *G. hirsutum SUTs*, we constructed an unrooted phylogenetic tree and compared coding and genomic sequences (Figure 3). The analyzed *SUTs* could be classified into three distinct groups; their structures showed great variation, with exon numbers ranging from 5 to 14. At the same time, most members of a given group had similar characteristics, differing only in the lengths of their introns. For example, all *SUT* genes in the SUT2 group contained 14 exons, which was approximately three times the number in other *SUT* genes, which had four or five exons. Within the SUT1 group, *GhSUT2A* and *GhSUT2D*, with 96.23% nucleotide sequence identity, and *GhSUT9A* and *GhSUT9D*, with 93.78% nucleotide sequence identity, had similar exon numbers and phase patterns. These results suggest that the exon/intron structure of *SUT* genes is associated with their evolutionary relationships and may reflect their functional conservation and divergence.

### Expression Patterns of *SUT* Genes in Different Tissues of *G. hirsutum*

**FIGURE 4:**
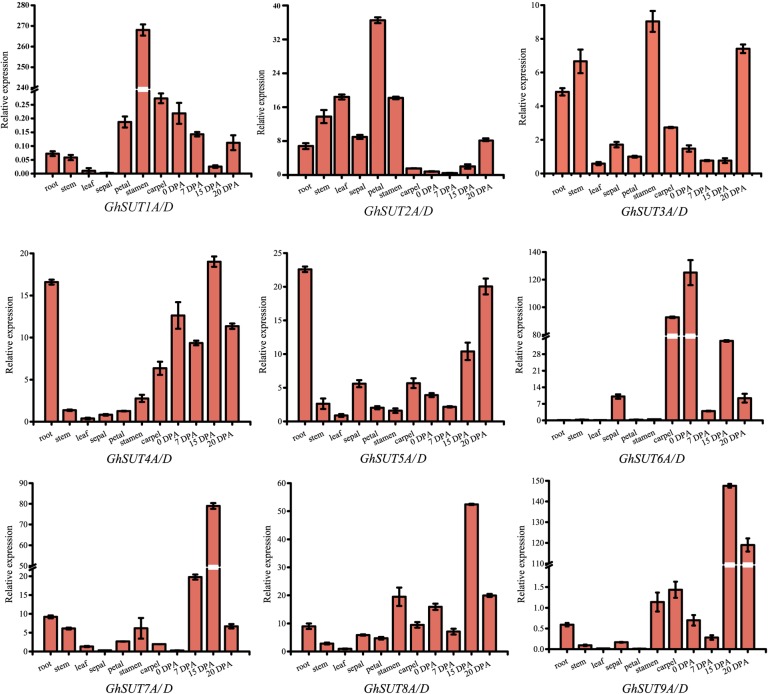
Tissues-specific expression profiles of *G. hirsutum SUT* genes. Expressions of nine paralogous *SUT* gene pairs were investigated in roots, stems, leaves, sepals, petals, stamens, carpels, ovules at 0 DPA, and fibers during different developmental stages by qRT-PCR. The *GhHis3* gene was used as an internal control. Error bars indicate the standard deviations of three independent experiments.

The expression pattern of a gene may reflect its biological function. In the present study, expression profiles of *G. hirsutum SUT* genes were investigated by qRT-PCR in several tissues, namely, roots, stems, leaves, sepals, petals, stamens, carpels, ovules at 0 DPA and fibers at 7 DPA, 15 DPA, and 20 DPA. Paralogs were difficult to distinguish by qRT-PCR because their sequences were highly similar. We therefore designed primers to amplify paralogs together (Figure 4). Overall, *SUT* genes exhibited diverse expression tendencies. Among the nine paralogous *SUT* gene pairs, *GhSUT7A/D*, *GhSUT8A/D*, and *GhSUT9A/D* were exclusively and highly expressed in 15 DPA fibers, which suggest that these genes play significant roles during cotton fiber elongation. *GhSUT1A/D* displayed a highly stamen-specific expression pattern. *GhSUT2A/D* was preferentially expressed in petals, while *GhSUT3A/D* was relatively highly expressed in roots, stems, stamens, and 20 DPA fibers. Transcripts of *GhSUT4A/D* and *GhSUT5A/D* were not only detected in fibers, but were also highly abundant in roots. Finally, *GhSUT6A/D* displayed higher expression levels in carpels and 0 DPA ovules.

### Abiotic Stress-Induced Expression Profiles of *G. hirsutum SUT* Genes

**FIGURE 5:**
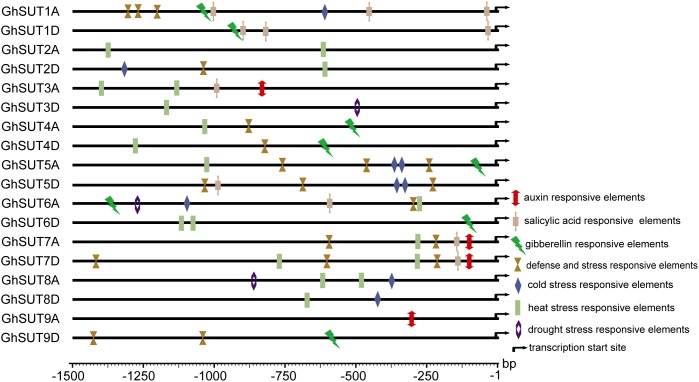
Putative *cis*-acting regulatory elements related to stress and hormone response in promoters of *G. hirsutum SUT* genes.

**FIGURE 6:**
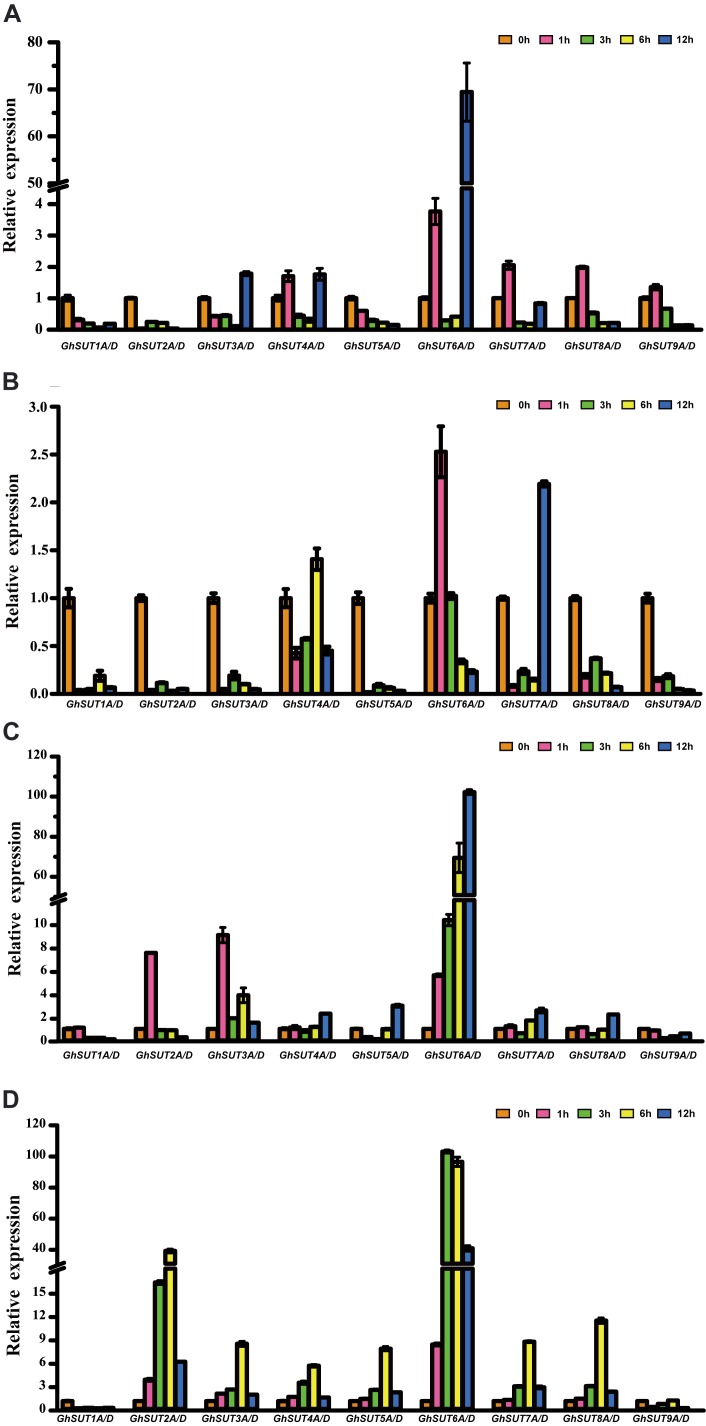
Expression analysis of *G. hirsutum SUT* genes by qRT-PCR under different abiotic treatments: **(A)** cold; **(B)** heat; **(C)** drought; **(D)** salt. Transcript levels of each gene were calculated by the 2^-ΔΔCt^ method and expressed relative to non-stressed values (0 h) set to 1. *GhHis3* was used as a housekeeping gene. Error bars represent the standard deviation of three independent experiments.

During growth and development, many plants are affected by various environmental conditions, such as exposure to low and high temperatures, high salinity, and drought. Previous studies have indicated that the plant *SUT* gene family is involved in abiotic and biotic stress responses (Frost et al., 2012; Jian et al., 2016; Liao et al., 2016; Xu et al., 2018). At the same time, multiple putative stress *cis*-acting regulatory elements were predicted in the promoters of *G. hirsutum SUT* genes in our study (Figure 5). We therefore profiled the expressions of all nine *SUT* paralogous gene pairs in *G. hirsutum* by qRT-PCR under four types of stress, namely, cold, heat, drought, and salinity (Figure 6). In general, the *SUT* genes exhibited variations in response to one or more stresses. Only a few genes were up-regulated at some treatment time point under heat stress, which suggest that most of these *SUT* genes are insensitive to high temperature and have complex regulatory mechanisms in response to heat stress. After cold treatment, *GhSUT6A/D* showed the greatest variation. *GhSUT4A/D* and *GhSUT7A/D* were up-regulated after 1 h of treatment, and their expressions then declined until 6 h of treatment and increased once more after 12 h of treatment. Transcripts of *GhSUT8A/D* and *GhSUT9A/D* were up-regulated after 1 h of constant cold treatment and were then down-regulated. After 1 h of drought stress, *GhSUT2A/D* and *GhSUT3A/D* genes were dramatically induced, and they were down-regulated after 3 h of treatment, up-regulated after 6 h, and down-regulated within 12 h of treatment. Expressions of *GhSUT4A/D*, *GhSUT7A/D*, and *GhSUT8A/D* were increased early during drought treatment, decreased at 3 h, and then gradually up-regulated during treatment until 12 h. Transcript levels of *GhSUT6A/D* were significantly increased during continued drought stress, whereas *GhSUT5A/D* and *GhSUT9A/D* were down-regulated at early time points in the treatment. Interestingly, seven out of nine *SUT* gene pairs were gradually up-regulated until 6 h of salt treatment and then showed a decrease at 12 h of treatment. Transcript levels of *GhSUT9A/D* decreased after 1 h of treatment, but increased from 3 to 6 h and then declined after 12 h. These results further suggest that these up-regulated *SUT* genes are involved in signaling pathways related to abiotic stress response during plant growth and development.

### Expression Analysis of *G. hirsutum SUT* Genes Exposed to Different Exogenous Phytohormones

**FIGURE 7:**
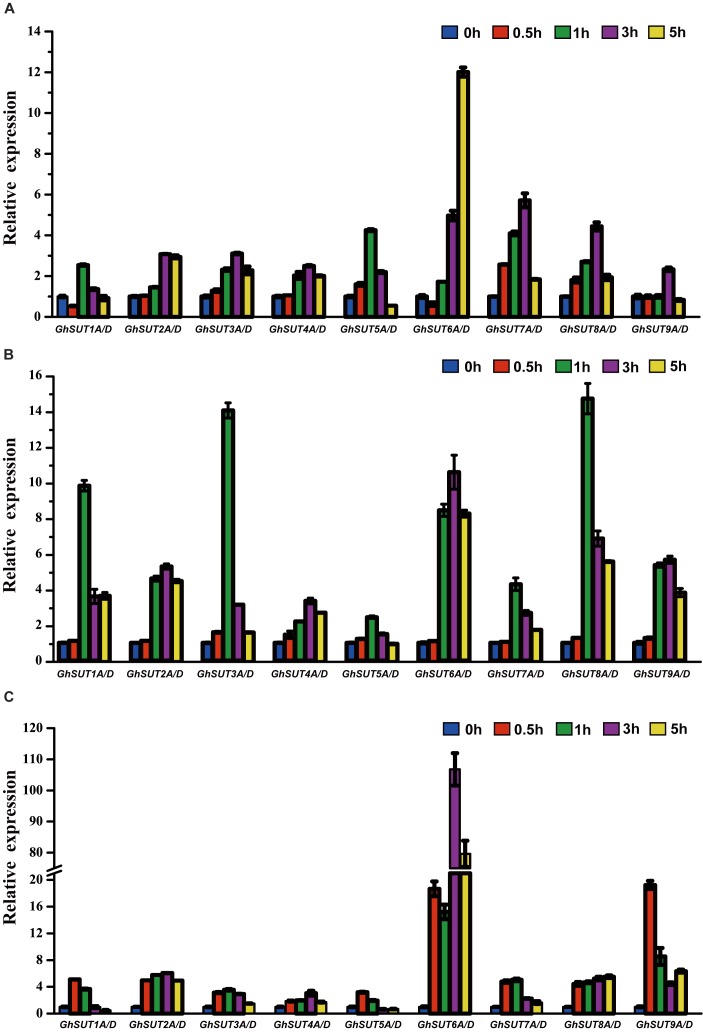
Relative transcriptional expression levels of *G. hirsutum SUT* genes based on qRT-PCR under different plant hormones treatments: **(A)** IAA; **(B)** GA; **(C)** SA. Transcript levels of each gene were calculated by the 2^-ΔΔCt^ method and expressed relative to the non-stressed values (0 h) set to 1. *GhHis3* was used as a housekeeping gene. Error bars represent the standard deviation of three independent experiments.

Our analysis of *cis*-regulatory elements in *G. hirsutum SUT* promoters, also revealed that most *G. hirsutum SUT* genes contained hormone stress-responsive *cis*-regulatory elements in their promoter regions (Figure 5). To further investigate the roles of *SUT* genes in response to plant hormones, we used qRT-PCR to examine the differential regulation of nine paralogous *SUT* gene pairs by IAA, GA, and SA stresses (Figure 7). After treatment with IAA, five *SUT* genes (*GhSUT2A/D*, *GhSUT3A/D*, *GhSUT4A/D*, *GhSUT7A/D*, and *GhSUT8A/D*) were significantly up-regulated until 3 h of treatment, with their expressions then decreasing at 5 h. Transcript levels of *GhSUT5A/D* gradually increased until 1 h of IAA treatment and then decreased from 3 to 5 h. Expression levels of *GhSUT6A/D* were slightly down-regulated at 0.5 h and then remained elevated during continued IAA stress. While *GhSUT9A/D* was not obviously altered at early time points in the IAA treatment, their expressions were significantly increased after 3 h of treatment, followed by a decline at 5 h. At different time points during GA treatment, meaningful differences were observed. Overall, *G. hirsutum SUT* genes generally exhibited two different expression trends. In particular, five genes (*GhSUT1A/D*, *GhSUT3A/D*, *GhSUT5A/D*, *GhSUT7A/D*, and *GhSUT8A/D*) were prominently induced until 1 h of treatment and then down-regulated for the remaining treatment period. Expressions of the other four *SUT* genes gradually increased during treatment until 3 h and then decreased after 5 h of treatment. Under SA treatment, *SUT* genes displayed diverse expression patterns. *GhSUT1A/D* and *GhSUT5A/D* were initially up-regulated and then down-regulated, while *GhSUT2A/D* and *GhSUT4A/D* transcript levels gradually increased and peaked at 3 h. Expressions of *GhSUT3A/D* and *GhSUT7A/D* slowly increased until 1 h of SA treatment, but these genes were then down-regulated for the remainder of the treatment. Surprisingly, transcript levels of *GhSUT6A/D* fluctuated greatly. The expression of this gene pair was instantly induced at 0.5 h, mildly decreased at 1 h, increased highly significantly after 3 h of treatment, and again reduced at 5 h. In contrast, *GhSUT8A/D* was strongly induced throughout the entire treatment period. *GhSUT9A/D* expressions showed a sharp increase after 0.5 h of treatment, around 20-fold higher than those under untreated conditions, and subsequently decreased until 3 h and then slowly increased beginning at 5 h of treatment.

## Discussion

*SUT* genes have been characterized in various plant species, including Arabidopsis (Srivastava et al., 2008; Li et al., 2012), rice (Aoki et al., 2003; Sun et al., 2010), maize (Usha, 2015), hexaploid wheat (Deol et al., 2013), *Medicago truncatula* (Doidy et al., 2012), *Populus* (Frost et al., 2012), cacao (Li et al., 2014b), and *Saccharum* (Zhang et al., 2016). In addition, recent findings have demonstrated that SUT proteins play crucial roles in cotton fiber elongation (Ruan et al., 2001; Zhang et al., 2017). No related studies, however, have focused on the genome-wide characterization and stress responses of this family in cotton. Comprehensive identification and analysis of the *SUT* gene family in cotton is needed to understand the evolution and functional role of this gene family as a foundation for future research.

### Conservation and Divergence of *G. hirsutum SUT* Genes During Evolution

SUTs, which mediate sucrose translocation from source tissues to sink cells, are characterized by 12 transmembrane-spanning domains in the cytoplasm (Aoki et al., 2003; Usha, 2015). Previous studies have revealed that a histidine residue in the extracellular loop is involved in sucrose binding and transportation (Deol et al., 2013; Zhang et al., 2013). Consistent with this earlier report, we also observed this structure in *G. hirsutum*, which implies that the 12 transmembrane-spanning domains and the histidine residue are highly conserved across different species (Supplementary Figure S1). Additionally, the dileucine-like motif LXXLL is present in the cytoplasmic N-terminal of group SUT4 members and facilitates the storage and transport of sucrose to the vacuolar membrane (Deol et al., 2013; Zhang et al., 2013). In our study, however, the motif LRQLX was located at this position, which differed by several amino acid residues compared with reported SUTs, suggesting that gene locus mutations and chromosomal recombinations and rearrangements have caused sequences to diverge after gene duplication events during upland cotton evolution. Exon/intron characteristics provide significant information on the evolution of a gene family (Lynch, 2002; Del Campo et al., 2013). In our study, exon numbers and structural patterns of *SUT* genes in the same group were highly similar, which suggested that genes in the same subfamily shared a common ancestor and have similar biological functions.

Biological evolution has played an important role in the history of life during the 4.6 billion years of the Earth’s existence. A- and D-genome diploid cotton species, originating, respectively, in Africa and Mexico, diverged approximately 5–10 MYA (Paterson et al., 2012). *G. hirsutum*, an allotetraploid species derived from the hybridization and chromosome doubling of an ancestral A-genome species resembling *G. arboreum* (A2) and a progenitor D-genome species resembling *G. raimondii* (D5), emerged approximately 1–2 MYA (Paterson et al., 2012; Li et al., 2015).

On the basis of phylogenetic relationships, previous authors have categorized plant *SUT* genes in two different ways, dividing them into five groups (SUT1, SUT2, SUT3, SUT4, and SUT5) (Kühn and Grof, 2010; Deol et al., 2013; Milne et al., 2013; Li et al., 2014b; Zhang et al., 2016) or alternatively three types (I, II, and III) (Aoki et al., 2003; Reinders et al., 2012; Zhang et al., 2013; Xu et al., 2018). In regard to the two classifications, we found that group SUT1 corresponds to type I, SUT2 belongs to type II, and SUT3, SUT4, and SUT5 are included in type III. The results of our study are consistent with the first classification scheme, which is more applicable to dicots and monocots (Figure 1). As SUT3 and SUT5 are specific to monocots, all 36 *SUT* genes from *G. arboreum*, *G. raimondii*, and *G. hirsutum* fall into SUT1, SUT2, and SUT4. Interestingly, only one member each of SUT2 and SUT4 are present in other analyzed plant species, whereas each of the three *Gossypium* species contain at least two *SUT* genes and may have undergone specific expansion caused by gene duplication. Our collinearity analysis revealed that two segmental duplication events have contributed to gene amplification in the two diploid cotton groups over the course of evolution (Figure 2). At the same time, the average divergence time of duplicated *SUT* genes in the two diploid cotton was estimated as 69.98 MYA, which implies that segmental duplications events may have occurred before the divergence of the A- and D-genome diploid and thus lead to the expansion of SUT2 and SUT4 groups in *G. arboreum* and *G. raimondii* (Table 2 and Supplementary Table S5). After natural polyploidization, the *SUT* loci were transferred from the two diploid genomes to the subgenomes of new tetraploid cotton indicated *GhSUTs* were conserved and subjected to strong purifying selection pressure. Consequently, segmental duplication and polyploidy have been the predominant contributors to the expansion of *SUT* genes in cotton, which is similar to findings reported for SOD and SWEET gene families (Wang et al., 2017; Li et al., 2018). Although evidence for tandem duplication was not found in our study, that process is also a basic driving force for gene expansion in genomic evolution (Flagel and Wendel, 2009). At the same time, phylogenetic analysis has revealed a close relationship between cacao and cotton, both of which belong to Malvaceae and have diverged from a common ancestor (Wang et al., 2012; Li et al., 2014a).

### Functional Role of *G. hirsutum SUT* Genes

Gene expression profiling can provide crucial clues to gene functions. We investigated the expressions of nine paralogous gene pairs in 11 different tissues of *G. hirsutum* by qRT-PCR. Most of the analyzed *SUT* genes were predominantly expressed in floral and fiber development processes. For example, *GhSUT1A/D* and *GhSUT2A/D* had very high expression levels in floral organs. The mutant of their ortholog *AtSUC9* is also abundantly expressed in flowers and promotes flowering under short-day conditions in Arabidopsis (Sivitz et al., 2007), which implies that *GhSUT1A/D* and *GhSUT2A/D* had functions in floral development similar to those of *AtSUC9*. In addition, *GhSUT4A/D*, *GhSUT7A/D*, *GhSUT8A/D*, *GhSUT9A/D*, *GhSUT3A/D*, and *GhSUT5A/D* were significantly expressed in 15 DPA and 20 DPA fibers. Only *GhSUT6A/D* displayed high expression levels during cotton fiber initiation. As is well known, fibers are the main product of cotton and the focus of genetic breeding research. A recent study has revealed that suppression of the expression of *GhSCP2D*, which encodes a putative sterol carrier protein, dramatically increases the expression of *GhSUT9A/D* (Locus ID: Gh_A13G1022/Gh_D13G1273) during fiber elongation stages, thus indicating that *SUT* genes play significant roles in cotton fiber cell elongation (Ruan et al., 2001; Zhang et al., 2017). Despite these findings, the molecular mechanism underlying the function of *G. hirsutm SUT* genes in fiber development is unclear and requires further investigation.

In addition to their association with plant growth, *SUT* genes are involved in the control of plant responses to abiotic stresses, biotic stresses, and phytohormones (Chincinska et al., 2008; Frost et al., 2012; Xu et al., 2018). For example, the *OsSUT2* gene is significantly up-regulated upon exposure to drought and salinity stress in rice (Ibraheem et al., 2011). In poplar, RNAi-*PtaSUT4* plants exhibit reduced rates of water uptake and delayed wilting in response to acute, short-term drought stress (Frost et al., 2012). Over-expression of the *NtSUT1* gene can alleviate inhibition of root elongation and confer higher growth capacity in aluminum-treated tobacco cells (Sameeullah et al., 2013; Kariya et al., 2017). *StSUT4* expression in wild-type potato plants is prominently induced by treatment with GA_3_ and the ethylene precursor ethephon (Chincinska et al., 2008). Furthermore, the *BnSUC1-2* gene is dramatically induced by SA, GA, and heat treatments in oilseed rape (Jian et al., 2016). In the present study, we analyzed *G. hirsutum SUT* gene expression patterns in response to four different abiotic and three phytohormone stress treatments. We found that most *SUT* genes were up-regulated under salt treatment (Figure 6). Physiologically, salinity, mainly from NaCl, can directly affect the absorption of water and nutrients, in turn reducing crop growth and yield. Although no salt-response elements were detected in *SUT* promoter regions, our results suggest that *SUTs* play essential roles in salt response. In a previous study, *G. hirsutum SUT* genes under drought stress exhibited various expression patterns consistent with those of *SUTs* in three *Saccharum* species (Zhang et al., 2016), and this phenomenon was also observed in our study. These results suggest that *SUT* genes are involved in distinct regulatory networks and have diverse functions in response to drought treatment. In addition, expression levels of almost all *SUT* genes were induced by different phytohormone treatments (Figure 7), which indicates that these genes likely participate in phytohormone-related signal responses. Notably, hormone stress-responsive *cis*-regulatory elements were found in most *G. hirsutum SUT* promoters (Figure 5), which provides further support for the likelihood that *G. hirsutum SUT* genes have phytohormone stress tolerance-related functions. In particular, the *GhSUT6A/D* gene pair of group SUT1 was intensively up-regulated under abiotic and phytohormone stresses compared with other *SUT* genes. Because few studies have been performed on stress-related functions of *GhSUT6* orthologs in Arabidopsis and rice, the putative stress response function of *GhSUT6* should be a focus. Taken together, these results provide novel clues regarding *G. hirsutum SUT* genes enhancement of tolerance to various stresses. Further analyses are needed to explore the possible relationship between sucrose transport and the physiological functions of *G. hirsutum SUT* genes triggered by different stresses.

## Conclusion

In this study, we performed a genome-wide analysis of *SUT* gene family members in *G. hirsutum*, including their classification, structure, evolutionary relationships, chromosomal location, expression patterns in diverse tissues, and transcriptional changes in response to a range of abiotic stresses and exogenous phytohormones. First, 18 *SUT* genes were identified in *G. hirsutum* and classified into three groups according to their phylogenetic relationships. *G. hirsutum SUT* genes within the same group displayed similar exon/intron characteristics. In addition, homologous genes in At and Dt subgenomes of the tetraploid cotton *G. hirsutum* and the two diploid cottons *G. arboreum* and *G. raimondii* exhibited one-to-one relationships. Furthermore, we found that the duplicated genes in the three cotton species have evolved through purifying selection, which suggest that *SUT* genes are highly conserved in these *Gossypium* species. Second, expression analyses in different tissues indicated that *SUT* genes may play significant roles in cotton fiber elongation. Third, analyses of *cis*-acting regulatory elements in promoter regions and expression profiling under different abiotic stress and exogenous phytohormone conditions implied that *SUT* genes, especially *GhSUT6A/D*, may participate in plant responses to diverse abiotic stresses and phytohormones. Our findings lay a foundation for future studies of the functions of *SUT* genes in plant stress response. In addition, these results may aid the breeding of new cotton varieties with enhanced stress tolerance and accelerate cotton genetic research.

## Author Contributions

DY, XM, and WL conceived and designed the research. KS, ZR, FZ, CS, and XZ performed the experiments. XP, YL, and KH prepared the materials. WL, KS, ZR, and ZW analyzed the data. WL and ZR wrote the paper. DY, XM, and KS revised the manuscript. All authors read and approved the final manuscript.

## Conflict of Interest Statement

The authors declare that the research was conducted in the absence of any commercial or financial relationships that could be construed as a potential conflict of interest.

## Footnotes

1 http://rice.plantbiology.msu.edu/index.shtml

2 https://phytozome.jgi.doe.gov/pz/portal.html#!info?alias=Org_Sbicolor

3 https://phytozome.jgi.doe.gov/pz/portal.html#!info?alias=Org_Bdistachyon

4 cocoa-genome-hub.southgreen.fr/download

5 http://www.genoscope.cns.fr/externe/GenomeBrowser/Vitis/

6 ftp://ftp.solgenomics.net/genomes/Solanum_lycopersicum/annotation/

7 http://www.arabidopsis.org

8 http://pfam.xfam.org/search#searchBatchBlock

9 http://chibba.agtec.uga.edu/duplication/mcscan/

10 https://www.ncbi.nlm.nih.gov/tools/primer-blast/index.cgi?LINK_LOC=BlastHome

## Supplementary Material

FIGURE S1 Alignment of amino acid sequences of all deduced SUTs in *G. hirsutum*. The 12 transmembrane domains are shown in bold and designated as Ito XII. The marked consensus sequence is derived from the highly conserved region of known functional plant SUTs, where 1 = I, L or V. The putative vacuolar targeting dileucine motif is shown in a red box.

FIGURE S2 Chromosomal distribution of *SUT* genes in *G. hirsutum*. The red dotted lines link paralogs located on At and Dt subgenomes. A megabase is provided.

TABLE S1 Sequences of qRT-PCR primers used to amplify nine *SUT* paralogous gene pairs in *G. hirsutum* and the *GhHis3* internal reference gene.

TABLE S2 Details on *G. raimondii* and *G. arboreum SUT* genes.

TABLE S3 Information on *SUT* genes in other species used in the study.

TABLE S4 The distribution of *SUT* family members in three monocot and seven dicot species.

TABLE S5 Ka/Ks analysis of duplicated *SUT* genes in *G. arboreum* and *G. raimondii*.
