# Supplementary figures and images for: Molecular Evolution and Stress and Phytohormone Responsiveness of SUT Genes in Gossypium hirsutum
Source: Front Genet. 2018 Oct 23;9:494. doi: 10.3389/fgene.2018.00494 (PMC6205988; doi:10.3389/fgene.2018.00494)

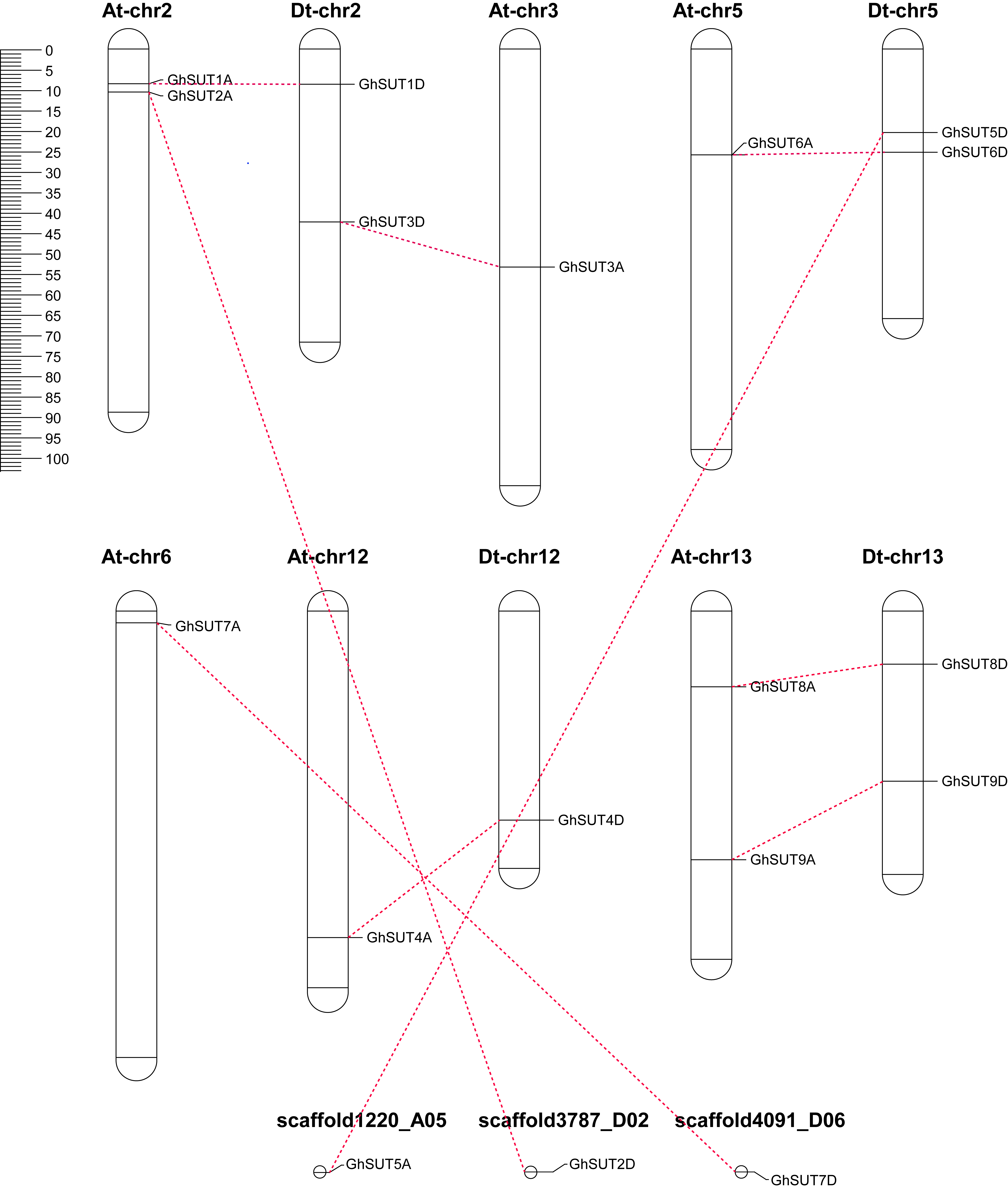

Supplement: FIGURE S2 — Chromosomal distribution of SUT genes in G. hirsutum. The red dotted lines link paralogs located on At and Dt subgenomes. A megabase is provided. [file Image_2.TIF]
