# Supplementary material for: Molecular Evolution and Stress and Phytohormone Responsiveness of SUT Genes in Gossypium hirsutum
Source: Front Genet. 2018 Oct 23;9:494. doi: 10.3389/fgene.2018.00494 (PMC6205988; doi:10.3389/fgene.2018.00494)
Supplement: TABLE S1 — Sequences of qRT-PCR primers used to amplify nine SUT paralogous gene pairs in G. hirsutum and the GhHis3 internal reference gene. [file Table_1.DOCX]

**Table S1.** Sequences of qRT-PCR primers used to amplify nine *SUT* paralogous gene pairs in *G. hirsutum* and the *GhHis3* internal reference gene.

| **Gene name** | **Forward Primer (5′–3′)** | **Reverse Primer (5′–3′)** |
| --- | --- | --- |
| *GhSUT1A/D* | ACAACAACAACTGCCTGTGAC | ATGATCCTCCTTCGCCTTCG |
| *GhSUT2A/D* | CCATTCGCTTTGGCATCCAT | CAGCACAAAAGCAGGCAAGT |
| *GhSUT3A/D* | TTTCAGCTGGTGCTAGTGGG | ACGAGTTGGCTGATCTGCTG |
| *GhSUT4A/D* | CTGTGAAGCCTGTGCCAATC | ATACGTGAGCTGGCTGGTTT |
| *GhSUT5A/D* | AGTGGGTGGTTTAAGGTTCTTCC | CCTGAGTGTTCAGGTCTCTCT |
| *GhSUT6A/D* | GGGCCTTGCAGTTATCCCTC | AGCTATAAAAGGGCGGCGAC |
| *GhSUT7A/D* | GAAGAGGGGCTTGAAGGTGG | ATCTCTCGACCCATCCAGTCA |
| *GhSUT8A/D* | CTAGTGGGAGTTGGCATCGG | GTGGAAGCGGAACCTCCTTA |
| *GhSUT9A/D* | CTTGACCCCTCCACCAACC | GACAGACCTTGACCGCCAC |
| *GhHis3* | TCAAGACTGATTTGCGTTTCCA | GCGCAAAGGTTGGTGTCTTC |
