# Supplementary material for: Molecular Evolution and Stress and Phytohormone Responsiveness of SUT Genes in Gossypium hirsutum
Source: Front Genet. 2018 Oct 23;9:494. doi: 10.3389/fgene.2018.00494 (PMC6205988; doi:10.3389/fgene.2018.00494)
Supplement: TABLE S2 — Details on G. raimondii and G. arboreum SUT genes. [file Table_2.DOCX]

Table S2. Details on *G. raimondii* and *G. arboreum* *SUT* genes.

| **Gene name** | **Locus ID** | **Strand** | **Chromosome** | **Location** |
| --- | --- | --- | --- | --- |
| *GrSUT1* | Gorai.005G067000 | Minus | Chr05 | 7,140,821-7,142,991 |
| *GrSUT2* | Gorai.005G075300 | Minus | Chr05 | 8,507,050-8,511,715 |
| *GrSUT3* | Gorai.005G139700 | Minus | Chr05 | 36,761,745-36,768,216 |
| *GrSUT4* | Gorai.008G181900 | Plus | Chr08 | 45,974,622-45,980,482 |
| *GrSUT5* | Gorai.009G225500 | Plus | Chr09 | 17,620,555-17,629,835 |
| *GrSUT6* | Gorai.009G264400 | Plus | Chr09 | 21,877,171-21,881,089 |
| *GrSUT7* | Gorai.010G030700 | Plus | Chr10 | 2,632,227-2,638,425 |
| *GrSUT8* | Gorai.013G083700 | Minus | Chr13 | 11,732,584-11,738,576 |
| *GrSUT9* | Gorai.013G140900 | Minus | Chr13 | 37,938,557-37,942,124 |
| *GaSUT1* | Cotton_A_08716 | Minus | CA_chr5 | 50,338,669-50,340,810 |
| *GaSUT2* | Cotton_A_08806 | Minus | CA_chr5 | 52,463,102-52,465,223 |
| *GaSUT3* | Cotton_A_35711 ^a^ | Minus | CA_chr8 | 54,012,595-54,017,984 |
| *GaSUT4* | Cotton_A_06140 ^a^ | Minus | CA_chr6 | 67,908,492-67,913,725 |
| *GaSUT5* | Cotton_A_19524 | Plus | CA_chr10 | 22,077,730-22,086,154 |
| *GaSUT6* | Cotton_A_04554 ^a^ | Plus | CA_chr6 | 104,651,050-104,653,978 |
| *GaSUT7* | Cotton_A_27876 | Minus | CA_chr11 | 37,113,377-37,118,043 |
| *GaSUT8* | Cotton_A_27958 | Plus | CA_chr1 | 5,877,967-5,882,622 |
| *GaSUT9* | Cotton_A_36400 | Plus | CA_chr13 | 54,261,528-54,264,686 |

^a^ The coding sequences of genes are manually re-annotated.
