# Supplementary material for: Molecular Evolution and Stress and Phytohormone Responsiveness of SUT Genes in Gossypium hirsutum
Source: Front Genet. 2018 Oct 23;9:494. doi: 10.3389/fgene.2018.00494 (PMC6205988; doi:10.3389/fgene.2018.00494)
Supplement: TABLE S3 — Information on SUT genes in other species used in the study. [file Table_3.DOCX]

**Table S3.** Information on *SUT* genes in other species used in the study.

| **Species** | **Gene name** | **Locus ID** |
| --- | --- | --- |
| Arabidopsis | *AtSUC1* | AT1G71880 |
| Arabidopsis | *AtSUC2* | AT1G22710 |
| Arabidopsis | *AtSUC3* | AT2G02860 |
| Arabidopsis | *AtSUC4* | AT1G09960 |
| Arabidopsis | *AtSUC5* | AT1G71890 |
| Arabidopsis | *AtSUC6* | AT5G43610 |
| Arabidopsis | *AtSUC7* | AT1G66570 |
| Arabidopsis | *AtSUC8* | AT2G14670 |
| Arabidopsis | *AtSUC9* | AT5G06170 |
| Rice | *OsSUT1* | LOC_Os03g07480 |
| Rice | *OsSUT2* | LOC_Os12g44380 |
| Rice | *OsSUT3* | LOC_Os10g26470 |
| Rice | *OsSUT4* | LOC_Os02g58080 |
| Rice | *OsSUT5* | LOC_Os02g36700 |
| Sorghum | *SbSUT1* | Sobic.001G254000 |
| Sorghum | *SbSUT2* | Sobic.001G488700 |
| Sorghum | *SbSUT3* | Sobic.004G190500 |
| Sorghum | *SbSUT4* | Sobic.004G353600 |
| Sorghum | *SbSUT5* | Sobic.007G214500 |
| Sorghum | *SbSUT6* | Sobic.008G193300 |
| *Brachypodium distachyon* | *BdSUT1* | Bradi1g73170 |
| *Brachypodium distachyon* | *BdSUT2* | Bradi3g25477 |
| *Brachypodium distachyon* | *BdSUT3* | Bradi3g46790 |
| *Brachypodium distachyon* | *BdSUT4* | Bradi3g56740 |
| *Brachypodium distachyon* | *BdSUT5* | Bradi4g00320 |
| Cacao | *TcSUT1* | Tc02v2_p029980 |
| Cacao | *TcSUT2* | Tc08v2_p006780 |
| Cacao | *TcSUT3* | Tc08v2_p016720 |
| Cacao | *TcSUT4* | Tc08v2_p016740 |
| Cacao | *TcSUT5* | Tc08v2_p016750 |
| Cacao | *TcSUT6* | Tc10v2_p004190 |
| Tomato | *SlSUT1* | Solyc11g017010 |
| Tomato | *SlSUT2* | Solyc05g007190 |
| Tomato | *SlSUT4* | Solyc04g076960 |
| Grape | *VvSUT1* | GSVIVT01009254001 |
| Grape | *VvSUT2* | GSVIVT01020031001 |
| Grape | *VvSUT3* | GSVIVT01034881001 |
