# Supplementary material for: Molecular Evolution and Stress and Phytohormone Responsiveness of SUT Genes in Gossypium hirsutum
Source: Front Genet. 2018 Oct 23;9:494. doi: 10.3389/fgene.2018.00494 (PMC6205988; doi:10.3389/fgene.2018.00494)
Supplement: TABLE S4 — The distribution of SUT family members in three monocot and seven dicot species. [file Table_4.DOCX]

**Table S4.** The distribution of *SUT* family members in three monocot and seven dicot species.

| **Species** | **SUT1** | **SUT2** | **SUT3** | **SUT4** | **SUT5** | **Total** |
| --- | --- | --- | --- | --- | --- | --- |
| *G.* *hirsutum* | 8 | 6 | 0 | 4 | 0 | 18 |
| *G. arboreum* | 4 | 3 | 0 | 2 | 0 | 9 |
| *G. raimondii* | 4 | 3 | 0 | 2 | 0 | 9 |
| Arabidopsis | 7 | 1 | 0 | 1 | 0 | 9 |
| Rice | 0 | 1 | 2 | 1 | 1 | 5 |
| Sorghum | 0 | 1 | 2 | 1 | 2 | 6 |
| *Brachypodium distachyon* | 0 | 1 | 2 | 1 | 1 | 5 |
| Cacao | 4 | 1 | 0 | 1 | 0 | 6 |
| Tomato | 1 | 1 | 0 | 1 | 0 | 3 |
| Grape | 1 | 1 | 0 | 1 | 0 | 3 |
