# Supplementary material for: Molecular Evolution and Stress and Phytohormone Responsiveness of SUT Genes in Gossypium hirsutum
Source: Front Genet. 2018 Oct 23;9:494. doi: 10.3389/fgene.2018.00494 (PMC6205988; doi:10.3389/fgene.2018.00494)
Supplement: TABLE S5 — Ka/Ks analysis of duplicated SUT genes in G. arboreum and G. raimondii. [file Table_5.docx]

**Table S5.** Ka/Ks analysis of duplicated *SUT* genes in *G. arboreum* and *G. raimondii*.

| **Species** | **Paralogous pairs** | **Ka** | **Ks** | **Ka/Ks** | **Purifing selection** | **Duplicate** | **Age (MYA)** |
| --- | --- | --- | --- | --- | --- | --- | --- |
| *G. arboreum* | *GaSUT4/8* | 0.1267 | 0.3899 | 0.3248 | Yes | Segment | 74.98076923 |
|  | *GaSUT5/7* | 0.0852 | 0.3786 | 0.2251 | Yes | Segment | 72.80769231 |
| *G. raimondii* | *GrSUT4/8* | 0.0796 | 0.3095 | 0.2571 | Yes | Segment | 59.51923077 |
|  | *GrSUT5/7* | 0.0767 | 0.3776 | 0.2032 | Yes | Segment | 72.61538462 |
